# Characterisation of meat consumption across socio-demographic, lifestyle and anthropometric groups in Switzerland: results from the National Nutrition Survey menuCH

**DOI:** 10.1017/S136898002200101X

**Published:** 2022-11

**Authors:** Linda Tschanz, Ivo Kaelin, Anna Wróbel, Sabine Rohrmann, Janice Sych

**Affiliations:** 1 Human Nutrition Laboratory, Department of Health Sciences and Technology, ETH Zurich, Switzerland; 2 Institute of Computational Lifesciences, ZHAW School of Life Sciences and Facility Management, Waedenswil, Switzerland; 3 Division of Chronic Disease Epidemiology, Epidemiology, Biostatistics and Prevention Institute, University of Zurich, Hirschengraben 84, 8001 Zurich, Switzerland; 4 Institute of Food and Beverage Innovation, ZHAW School of Life Sciences and Facility Management, Waedenswil, Switzerland

**Keywords:** Meat consumption, Dietary survey, Meat determinants, Switzerland

## Abstract

**Objective:**

Characterising meat consumption in Switzerland across socio-demographic, lifestyle and anthropometric groups.

**Design:**

Representative national data from the menuCH survey (two 24-hour dietary recalls, anthropometric measurements and a lifestyle questionnaire) were used to analyse the total average daily intake of meat and main meat categories. Energy-standardised average intake (g/1000 kcal) was calculated and its association with 12 socio-demographic, lifestyle and anthropometric variables was investigated using multivariable linear regression.

**Setting:**

Switzerland.

**Participants:**

Totally, 2057 participants aged 18–75 years.

**Results:**

Average total meat intake was 109 g/d, which included 43 g/d of processed meat, 37 g/d of red meat and 27 g/d of white meat. Energy-standardised meat intake was highest for men, the Italian-language region and the youngest age group (18–29 years). Regression results showed significantly lower total meat and red meat consumption (g/1000 kcal) for women than men. However, there were no sex-specific differences for white meat. Total meat and white meat consumption were positively associated with the 18–29 age group, compared with 30–44 years, non-Swiss compared with Swiss participants and one-parent families with children compared with couples without children. Consumption of all categories of meat showed positive associations for BMI > 25 kg/m^2^ compared with BMI 18·5–25 kg/m^2^ and for French- and Italian-language regions compared with German-language region.

**Conclusion:**

The current study reveals that there are significant differences in the amounts and types of meat consumed in Switzerland, suggesting that evidence-based risks and benefits of these categories need to be emphasised more in meat consumption recommendations.

Meat is known to contribute significant amounts of protein, minerals and vitamins to the human diet^(1)^. However, in large meta-analyses of prospective studies, certain meat categories, in particular processed meat (PM) and unprocessed red meat, have been associated with increased risk of several non-communicable diseases such as CVD^(2,3)^, specific cancer types such as colorectal cancer^(4)^ and type-2 diabetes^(3)^.

In addition, these results indicate that a consumption level of more than 50–100 g/d of red meat is considered to be associated with health risks^(2,4)^. Several nutritional societies have revised their recommendations to limit the consumption of PM and red meat types^(5–7)^. This information presents an interesting contrast to current dietary recommendations in Switzerland where the Swiss Food Pyramid presents meat alongside with fish and plant-based protein sources, such as tofu, on the fourth pyramid level^(8)^. Since 2011, adults have been advised to consume 100–120 g of these products daily. To meet this recommendation, consumption of two to three portions of meat (including poultry) weekly, but no more than one portion of PM per week, are recommended. Conducted every five years and since 1992, the Swiss Health Survey assesses the consumption frequency of selected food items using an abbreviated food list that includes the combined intakes of meat and meat products. According to this survey, from 1992 to 2017, the percentage of the Swiss population adhering to the recommendation of only consuming meat two to three times per week increased from 39 % to 47 %.^(9)^. Additionally, food balance sheet data from 2007 to 2018 have shown a declining trend in annual consumption of all meat types except poultry-based products^(10)^. The only other sources of quantitative meat consumption data in Switzerland are reports about individual regions and selected demographic groups^(11,12)^.

In Switzerland, there is also a lack of national data that connect socio-demographics, lifestyle and meat consumption. The drivers behind meat consumption are complex and vary considerably between countries. From a public health and sustainability perspective, this knowledge is valuable for the development of more effective strategies to influence the habits of population subgroups with high meat consumptions^(13)^. We have previously shown differences in the consumption of PM^(14)^ and in types of meat consumers (i.e. non-consumers *v*. low, moderate and high consumers)^(15)^ in the Swiss population based on socio-economic, lifestyle and anthropometric factors. To date, no study has addressed whether and how these factors influence the consumption of unprocessed red meat and white meat in subgroups of the Swiss population.

In light of the above discussions, the aim of the current study was to examine the meat consumption of the Swiss population, focusing on total meat consumption, and in particular unprocessed red and white meat. Additionally, it will investigate how meat consumption is associated with selected socio-demographic, lifestyle and anthropometric factors.

## Materials and methods

In the current analysis, data from menuCH, a population-based, cross-sectional National Nutrition Survey, were analysed to characterise meat consumption in Switzerland. The menuCH study included 2057 participants (response rate of 38 %), who were interviewed about their dietary habits in 2014/2015. Adults between 18 and 75 years of age were randomly drawn from a stratified sample intended to be representative of the seven Swiss regions (Lake Geneva Regions, Mittelland, Northwestern Switzerland, Zurich, Eastern Switzerland, Central Switzerland and Ticino) and of sex and age of the population (five age groups: 18–29, 30–39, 40–49, 50–64 and 65–75)^(16)^. Diet was assessed using two 24-hour dietary recalls (24HDR). The first 24HDR interview was conducted in person and the second was completed by telephone. During the first interview, anthropometric measurements (height, weight and waist circumference) were recorded according to the MONICA protocol from the WHO and used to calculate the BMI^(17)^. When body measurements were not possible (fourteen pregnant women, thirteen lactating and seven other participants), self-reported weight or height was used. Food consumption of the participants was recorded using the trilingual Swiss version (0·2014·02·27) of GloboDiet® software (formerly EPIC-Soft®, International Agency for Research on Cancer IARC, Lyon, France^(18)^ adapted for Switzerland by the Federal Food Safety and Veterinary Office, Bern, Switzerland). Data cleaning was carried out using an updated version of the software (2015·09·28). The consumption data were linked to the Swiss Food Composition Database^(19)^. The menuCH questionnaire contained questions on nutrition and dietary habits as well as socio-demographic and economic factors. The short version of the International Physical Activity Questionnaire was used to assess the physical activity^(20)^.

We used the consumption data from the two 24HDR interviews and selected socio-demographic and lifestyle information from the menuCH questionnaire to quantify and characterise meat consumption. According to a model used in our earlier work on menuCH data,^(14)^,^(15)^ the following socio-demographic, anthropometric and lifestyle variables were selected a priori and investigated in relation to meat consumption: sex (male, female), language region (German, French and Italian), age group (18–29, 30–44, 45–59 and 60–75 years), BMI category (< 18·5, 18·5–< 25·0, 25·0–< 30·0 and ≥ 30 kg/m^2^), nationality (Swiss only, Swiss with dual citizenship and non-Swiss), gross household income (< 6000, 6000–13 000 and > 13 000 Swiss Francs/month), education (primary-, secondary- and tertiary-level education), smoking status (never, former and current), household status (living alone, adult living with parents, one-parent family with children, couple without children, couple with children and others), physical activity level (low, medium and high), health status (very poor to medium, good to very good) and currently following a weight-loss diet (yes, no).

In our analysis, we differentiated between unprocessed red meat and unprocessed white meat, which corresponds to meat from mammals and poultry, respectively. All meat refers to ‘meat and meat products’ as defined in Globodiet® with the addition of meat from bolognaise sauce and the removal of meat substitutes (Fig. S1) and represents all meat consumption by menuCH participants. The groups ‘PM and sausages’, ‘bolognaise sauce’ and ‘meat skewer’ from Globodiet® were categorised as PM^(14)^. Unprocessed meat (UPM) included all meat not categorised as PM, and UPM was categorised into sub-categories of white, red and other UPM. Offal was included with the respective meat type: red meat included meat from mammals and its offal; white meat included poultry and its offal and other UPM included meat that was not specified by the participants and mixed meat. The total consumption of the most consumed UPM types (see online supplementary material, Supplemental Fig. S2) was also quantified according to the following sub-categories: beef, pork, lamb and veal from the red meat category and poultry from the white meat category. In this categorisation, all quantities of offal were combined to form a separate meat type (offal). The remaining UPM consisted of rarely consumed meat types such as game, horse, rabbit and goat as well as any meat consumption, which was not clearly specified by study participants, i.e. unspecified meat types.

Only participants who completed both 24HDR interviews were included in the analysis (*n* 2057). Mean daily meat consumption is presented in grams per day with the standard error of the mean (g/d; sem). To correct for sampling design and nonresponse, survey weighting factors such as sex, age, major areas of Switzerland, marital status, household size and nationality were applied to the means of all results. The means for consumption data were also weighted for season and weekday to account for uneven collection of 24HDR over the year and week.^(21)^. To facilitate comparisons between demographic groups, energy-standardised meat consumption (g/1000 kcal, sem) was calculated for the main meat categories and used to investigate associations between meat consumption and socio-demographic, anthropometric and lifestyle factors (sex, language region, age, BMI, nationality, education, household status, income, physical activity, smoking, health status and following a weight-loss diet) using multivariable linear regression. Results were presented as coefficients with a 95 % CI of energy-standardised meat consumption. All analyses were performed with R-4·0·2, using the ggplot2, dplyr and plyr packages. To estimate missing values from the questionnaire, multivariate imputation by chained equations was performed using the R-package mice. The results were reported according to the STROBE-nut guidelines^(22)^.

## Results

All 2057 of the participants in our analysis lived in Switzerland, 933 were men and 1124 were women (Table 1). Once weighting factors had been applied to the survey data, the sample represented 4 627 878 residents of Switzerland, which were balanced between the sexes and of which 60 % were between 30 and 59 years old. The majority was German-speaking (69 %) and of Swiss nationality (61 %). Regarding lifestyle, 23 % of the study population were current smokers and 44 % were overweight or obese. In terms of education, 53 % of the participants had a tertiary degree.

**Table 1:** Description of the menuCH population and its processed meat, unprocessed meat, red meat and white meat consumers (*n* 2057)

	Total sample		PM consumers	UPM consumers	Red meat consumers	White meat consumers
	Crude	Weighted*	Weighted*	Weighted*	Weighted*	Weighted*
	%	%	%	%	%	%
Sex						
Men	45·4	49·8	54·9	52·9	55·1	50·2
Women	54·6	50·2	45·1	47·1	44·9	49·8
Language region†						
German	65·2	69·2	71·0	67·9	67·0	63·8
French	24·4	25·2	23·5	26·6	27·7	30·1
Italian	10·4	5·6	5·5	5·5	5·3	6·1
Age group‡						
18–29 years	19·4	18·8	18·0	19·3	15·9	24·2
30–44 years	25·9	29·8	31·0	30·5	30·9	31·7
45–59 years	30·4	29·8	30·1	29·5	29·6	28·9
60–75 years	24·3	21·6	20·9	20·7	23·6	15·2
BMI§						
Underweight (BMI < 18·5 kg/m^2^)	2·4	2·4	2·0	1·6	1·6	1·9
Normal (18·5 ≤ BMI < 25·0 kg/m^2^)	54·3	54·1	53·0	51·3	49·3	53·9
Overweight (25·0 ≤ BMI < 30·0 kg/m^2^)	30·5	30·6	31·5	32·3	33·5	31·2
Obese (BMI ≥ 30·0 kg/m^2^)	12·8	12·9	13·5	14·8	15·6	13·0
Nationality						
Swiss	72·5	61·4	62·7	59·4	62·4	53·0
Swiss binational	14·4	13·8	23·6	14·8	14·6	16·4
Non-Swiss	13·0	24·8	13·7	25·8	23·0	30·6
Education level						
Primary	4·3	4·7	4·4	5·0	4·5	6·0
Secondary	47·1	42·6	43·1	42·9	45·8	37·9
Tertiary	48·5	52·7	52·5	52·1	49·7	56·1
Household status						
Living alone	16·0	18·2	16·4	17·8	16·3	19·2
Adult living with parents	7·7	7·1	7·3	8·0	7·9	8·8
One-parent family with children	4·5	4·4	4·2	4·6	4·8	4·8
Couple without children	33·5	31·7	31·4	30·2	33·1	24·8
Couple with children	33·0	32·9	34·8	33·6	32·7	36·1
Others	5·3	5·7	5·9	5·8	5·2	6·3
Income						
< 6000	16·8	17·7	16·3	16·8	16·9	15·3
6000–13 000	40·9	39·8	40·8	39·7	39·5	38·4
> 13 000	13·9	14·9	16·0	14·8	14·8	16·6
Imputed^||^	28·4	27·6	26·9	28·7	28·8	29·7
Physical activity						
Low	14·7	12·9	17·0	15·7	16·7	14·4
Medium	22·1	22·7	21·2	21·8	21·7	23·4
High	40·2	40·3	40·6	40·1	38·2	41·4
Imputed^||^	23·0	24·2	21·2	22·4	23·4	20·8
Smoking status						
Never	44·5	42·9	40·9	42·0	41·0	42·9
Former smoker	33·5	33·7	34·5	33·2	33·6	31·7
Current smoker	22·0	23·4	24·6	24·8	25·4	25·4
Health status						
Very poor to medium	13·3	12·8	13·1	13·0	13·7	11·2
Good to very good	86·7	87·2	86·9	87·0	86·3	88·8
Currently on a diet						
Yes	5·6	5·5	4·7	6·4	6·2	7·1
No	94·4	94·5	95·3	93·6	93·8	92·9

PM, processed meat; UPM, unprocessed meat; CHF, Swiss francs.

* Weighted for sex, age, marital status, major area of Switzerland, household size and nationality.

† German-language region: cantons Aargau, Basel–Land, Basel–Stadt, Bern, Lucerne, St. Gallen, Zurich; French-language region: Geneva, Jura, Neuchâtel, Vaud; Italian-language region: Ticino.

‡ Self-reported age on the day of the first 24-hour dietary recall interview.

§ BMI was based on measured height and weight, or on self-reported estimations when measurements were not possible.

|| Multivariate imputation by chained equations was used for missing values; imputed values of < 0·4 % are not shown.

Eighty-nine percent of the menuCH study population reported that they consumed meat. When looking specifically at PM, UPM, red meat and white meat, the respective rate of consumption was 72 %, 69 %, 46 % and 35 %. The demographics of processed, unprocessed and red meat consumers were similar to the entire study population, whereas the demographics of white meat consumers showed some differences to other meat categories: there were equal numbers of female and male study participants, but there were higher numbers of participants from the youngest age group, with non-Swiss nationality, a tertiary-level education and with children (Table 1).

Table 2 shows that the mean daily meat consumption was 109 g/d, including 66 g/d UPM. The actual and energy-standardised meat consumption of men was higher than that of women by 58 g/d and 13 g/1000 kcal, respectively. In the French- and Italian-language regions, all meat and UPM consumption (actual and energy-standardised) was higher than in the German-language region. Actual and energy-standardised daily meat consumption decreased slightly over the age groups, while UPM consumption was highest in the 30–44 age group.

**Table 2:** Mean daily consumption of all meat and main sub-categories of unprocessed meat, red, white and other meat by total population, sex, language region and age group based on two 24-hour dietary recalls, *n* 2057 (g/d, g/1000 kcal)*

	All Meat			All Unprocessed Meat			Red Meat			White Meat			Other		
	Mean	sem	Mean	Mean	sem	Mean	Mean	sem	Mean	Mean	sem	Mean	Mean	sem	Mean
	(g/d)		(g/1000 kcal)	(g/d)		(g/1000 kcal)	(g/d)		(g/1000 kcal)	(g/d)		(g/1000 kcal)	(g/d)		(g/1000 kcal)
Total sample	108·9	2·0	49·9	66·2	1·6	31·2	36·9	1·2	17·1	26·8	1·1	13·0	2·5	0·3	1·1
Sex															
Men	138·2	3·3	56·5	83·0	2·8	34·6	48·2	2·2	19·8	31·3	1·9	13·5	3·5	0·6	1·4
Women	79·8	1·9	43·2	49·5	1·6	27·8	25·6	1·2	14·5	22·3	1·1	12·6	1·6	0·3	0·8
Language region†															
German	105·6	2·4	47	60·5	1·9	27·6	33·1	1·5	14·9	24·3	1·3	11·5	3·1	0·4	1·2
French	115·9	4·1	55·2	79·1	3·5	38·8	45·3	2·7	22·3	32·2	2·5	15·7	1·6	0·5	0·8
Italian	116·7	6·5	61·4	77·4	5·6	41·3	44·7	4·9	21·5	32·2	3·6	19·6	0·5	0·3	0·2
Age group‡															
18–29 years	123·8	5·0	53·9	73·8	4·1	33·5	32·0	2·8	14·1	38·2	3·1	17·4	3·5	0·7	1·9
30–44 years	117·4	4·1	51·7	74·6	3·5	33·4	41·4	2·7	18·2	29·9	2·3	14·0	3·2	0·8	1·1
45–59 years	103·8	3·4	48·7	63·3	2·8	31·0	37·0	2·2	18·0	23·9	1·8	12·1	2·5	0·6	0·9
60–75 years	91·0	3·3	45·4	51·9	2·5	26·5	34·5	2·2	17·1	16·6	1·5	9·0	0·8	0·2	0·4

sem, standard error of the mean.

* Weighted for sex, age, marital status, major area of Switzerland, household size, nationality, season and weekday.

† German-language region: cantons Aargau, Basel–Land, Basel–Stadt, Bern, Lucerne, St. Gallen, Zurich; French-language region: Geneva, Jura, Neuchâtel, Vaud; Italian-language region: Ticino.

‡ Self-reported age on the day of the first 24-hour dietary recall. Other unprocessed meat corresponds to unprocessed meat that was not further specified.

**Fig. 1:**
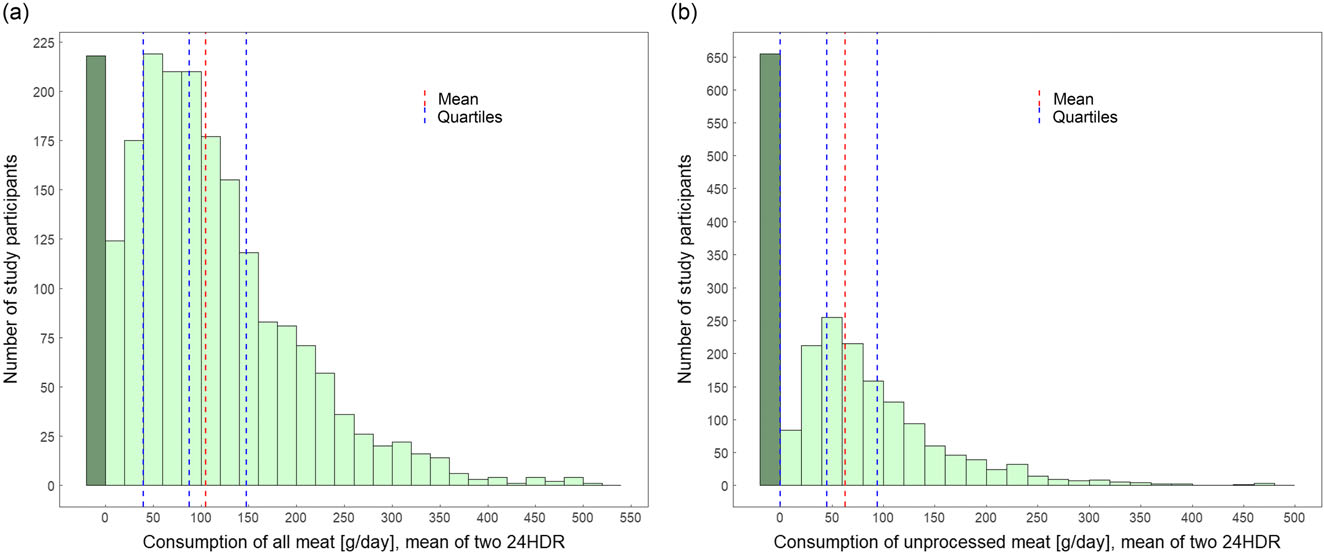
The histograms present the number of study participants and the consumption of all meat (a) and of unprocessed meat (b) by participants, using the mean of two 24-hour dietary recalls (g/d). For the sample, the dark green bar indicates no meat consumption; the red line indicates mean intake; and the blue lines are first, second and third quartiles. All data were weighted for sex, age, marital status, major area of Switzerland, household size, nationality, season and weekday (*n* 2057)

UPM consumption by sex, language region and age group showed similar variations between the demographic groups as for all meat (Table 2). An exception was that energy-standardised all-meat consumption seemed to decrease progressively over the age groups, whereas UPM consumption was similar for the three age groups (18–59 years) and slightly lower in the oldest age group. In the French- and Italian-language regions, higher amounts of UPM were consumed, 18–19 g/d and 11–13 g/1000 kcal, respectively, than in the German-language region. The histograms for all meat and UPM consumption, showing the distribution of daily mean consumption by the population, had a similar skewed shape but compared with all meat, higher number of participants did not consume UPM (Fig. 1).

Mean daily red meat consumption was 37 g/d, corresponding to 34 % of all meat consumption (Table 2). Men consumed approximately twice the amount of red meat as women. Absolute and energy-standardised red-meat consumption were similar in the French- and Italian-language regions, and both were higher than in the German-language region. The energy-standardised consumption of red meat did not suggest a decrease over the age groups, unlike all meat (Table 2).

The mean daily consumption of 27 g/d of white meat contributed 25 % to all meat consumption (Table 2). Mean energy-standardised white meat consumption was the same for men and women. White meat consumption in the French- and Italian-language regions was higher than that in the German-language region. The mean daily as well as the mean energy-standardised white meat consumption decreased over the age groups.

Table 3 and Supplemental Table S1 detail the consumption of the most common UPM types. The meat type consumed in highest quantities by the menuCH population was poultry (27 g/d – without poultry offal) followed by beef (16 g/d), pork (11 g/d), veal and lamb (3 g/d). Only a small amount of offal was consumed (1 g, all offal types combined). The remaining 6 g/d of UPM consisted of rarely consumed meat types such as game, horse, goat, rabbit and mixed meat products of UPM. Men consumed more of every UPM type than women (actual and energy-standardised consumption) except for poultry. Beef consumption in the Italian and the French-language regions was almost twofold higher than that of the German-language region. Pork consumption was lowest in the French-language region and a decrease was observed in the 30–44 age group and across the older age groups. The highest veal consumption was reported in the Italian region and the lowest consumption occurred in the French-language region. The highest lamb consumption was reported in the French-language region and in the oldest age group. Offal was also consumed the most in the French-language region and by study participants aged 45–59 years.

**Table 3:** Mean daily consumption of unprocessed meat sub-categories by total population, sex, language region and age group based on two 24-hour dietary recalls, *n* 2057 (g/d)*

	Poultry		Beef		Pork		Veal		Lamb		Offal		Remaining unprocessed meat	
	Mean (g/d)	sem	Mean (g/d)	sem	Mean (g/d)	sem	Mean (g/d)	sem	Mean (g/d)	sem	Mean (g/d)	sem	Mean (g/d)	sem
Total sample	26·6	1·1	15·6	0·8	11·3	0·7	3·3	0·3	2·7	0·3	1·0	0·2	5·7	0·5
Sex														
Men	31·1	1·9	19·4	1·3	14·6	1·2	4·3	0·6	3·4	0·6	1·5	0·4	8·7	1·0
Women	22·1	1·1	11·7	0·9	8·0	0·7	2·4	0·3	1·9	0·3	0·6	0·2	2·8	0·4
Language region†														
German	24·3	1·3	12·4	0·8	12·4	0·9	3·7	0·4	2·3	0·4	0·5	0·2	4·9	0·5
French	31·4	2·4	22·7	1·8	8·6	1·1	1·8	0·5	4·2	0·8	2·5	0·6	7·8	1·3
Italian	32·2	3·6	21·3	3·6	10·3	2·7	6·3	1·6	0·4	0·4	0·4	0·4	6·5	1·8
Age group‡														
18–29 years	38·0	3·1	14·1	1·8	10·4	1·7	2·6	0·6	2·1	0·6	0·2	0·1	6·3	1·1
30–44 years	29·5	2·2	18·1	1·7	13·7	1·5	3·4	0·7	2·6	0·6	1·2	0·5	6·0	1·0
45–59 years	23·7	1·8	14·7	1·3	11·1	1·2	3·9	0·7	1·7	0·6	1·5	0·4	6·8	1·1
60–75 years	16·4	1·5	14·5	1·5	9·0	1·2	3·2	0·6	4·6	0·8	0·8	0·3	3·5	0·7

sem, standard error of the mean.

* Weighted for sex, age, marital status, major area of Switzerland, household size, nationality, season and weekday.

† German-language region: cantons Aargau, Basel–Land, Basel–Stadt, Bern, Lucerne, St. Gallen, Zurich; French-language region: Geneva, Jura, Neuchâtel, Vaud; Italian-language region: Ticino.

‡ Self-reported age on the day of the first 24-hour dietary recall. Remaining unprocessed meat includes unspecified unprocessed meat and meat from animals not specifically listed in this table. Offal includes offal of poultry, beef, pork, veal, lamb and remaining unprocessed meat.

Taking confounding factors into account, the energy-standardised meat consumption of men was significantly higher than that of women, with the exception of white meat consumption (Table 4). Consumption of all meat categories differed significantly between the language regions and between the BMI groups. Higher energy-standardised meat consumption (all categories) was observed in the French and the Italian language regions than in the German-language region; in obese and overweight participants compared with those with a normal BMI, and in current smokers compared with those who have never smoked (only in the category of all meat). Additionally, factors such as age, nationality, education degree, household status and following a weight-loss diet were identified as possible determinants for meat-category consumption amounts.

**Table 4:** Associations of energy-standardised consumption of all meat, red meat and white meat with socio-demographic and lifestyle factors, by multivariable linear regression analysis, *n* 2057 (g/1000 kcal)*

	All meat (g/1000 kcal)		Red meat (g/1000 kcal)		White meat (g/1000 kcal)	
	Coefficients	95 % CI†	Coefficients	95 % CI†	Coefficients	95 % CI†
Sex						
Men	0	ref.	0	ref.	0	ref.
Women	−10·12	−13·60, –6·64	−3·90	−6·35, –1·51	−0·81	−3·01, 1·39
Language regions‡						
German	0	ref.	0	ref.	0	ref.
French	6·97	3·14, 10·79	7·40	4·74, 10·06	2·91	0·49, 5·33
Italian	11·90	4·70, 19·11	5·58	0·58, 10·59	7·02	2·45, 11·59
Age groups§						
18–29 years	7·05	1·41, 12·68	−2·80	−6·73, 1·08	8·37	4·81, 11·93
30–44 years	0	ref.	0	ref.	0	ref.
45–59 years	−4·64	−8·96, –0·31	−1·00	−3·99, 2·01	−1·52	−4·25, 1·22
60–75 years	−5·29	−10·63, 0·05	−0·80	−4·51, 2·89	−2·11	−5·49, 1·27
BMI categories||						
Underweight	−7·40	−18·31, 3·51	−4·90	−12·50, 2·65	−2·79	−9·69, 4·11
Normal	0	ref.	0	ref.	0	ref.
Overweight	12·95	9·09, 16·81	5·30	2·63, 8·00	3·93	1·48, 6·37
Obese	18·21	12·80, 23·63	5·80	2·04, 9·56	6·15	2·72, 9·58
Nationality						
Swiss only	0	ref.	0	ref.	0	ref.
Swiss binational	5·03	0·21, 9·87	2·03	−1·32, 5·37	1·97	−1·08, 5·02
Non-Swiss	6·86	2·24, 10·89	2·02	−0·85, 4·88	5·98	3·37, 8·59
Education level						
Primary	0·21	−7·89, 8·31	−8·04	−13·63, –2·45	4·63	−0·50, 9·76
Secondary	0	ref.	0	ref.	0	ref.
Tertiary	−8·60	−12·23, –4·98	−5·59	−8·09, –3·08	−0·86	−3·16, 1·44
Household status						
Living alone	2·79	−2·61, 8·19	−3·49	−7·20, 0·21	5·65	2·26, 9·05
Adult living with parents	−2·19	−9·99, 5·61	−1·61	−7·01, 3·78	−4·85	−9·79, 0·09
One-parent family with children	9·04	0·43, 17·65	4·68	−1·29, 10·65	6·13	0·68, 11·58
Couple without children	0	ref	0	ref.	0	ref.
Couple with children	5·03	0·67, 9·39	0·77	−2·26, 3·79	3·64	0·89, 6·40
Others	−0·21	−7·96, 7·55	−1·12	−6·50, 4·25	−2·03	−6·95, 2·89
Income (CHF/month)						
< 6000	−0·11	−5·87, 5·66	0·77	−2·84, 4·38	−1·15	−4·30, 2·00
6000–13 000	0	ref.	0	ref	0	Ref.
> 13 000	−0·36	−5·44, 4·72	−1·22	−5·08, 2·64	1·03	−2·69, 4·74
Physical activity level						
Low	0	ref	0	ref.	0	ref
Moderate	−3·61	−9·32, 2·09	−1·12	−5·34, 3·10	0·02	−3·28, 3·32
High	−3·26	−8·53, 2·01	−1·70	−5·76, 2·36	0·86	−2·44, 4·16
Smoking status						
Never smoked	0	ref.	0	ref.	0	ref.
Former smoker	0·69	−3·06, 4·44	−0·97	−3·55, 1·62	−0·33	−2·70, 2·03
Current smoker	4·40	0·04, 8·76	2·15	−0·87, 5·17	−0·64	−3·40, 2·11
Health status						
Very poor to medium	0·12	−5·13, 5·36	0·35	−3·29, 3·98	−0·82	−4·14, 2·49
Good to very good	0	ref.	0	ref.	0	ref.
Currently on a diet						
Yes	0·09	−7·02, 7·20	1·46	−3·48, 6·39	6·32	1·81, 10·82
No	0	ref.	0	ref.	0	ref.

CHF, Swiss francs.

* Results of the multivariable linear regressions were adjusted for all variables presented in this table and weighted for sex, age, marital status, major area of Switzerland, household size, nationality, season and weekday.

† CI: conﬁdence interval.

‡ German-language region included cantons: Aargau, Basel–Land, Basel–Stadt, Ben, Lucerne, St. Gallen, Zurich; French-language region: Geneva, Jura, Neufchâtel, Vaud and Italian-language region: Ticino.

§ Age corresponds to self-reported age on the day of the first 24-hour dietary recall.

|| BMI was based on measured height and weight, or on self-reported estimations when measurements were not possible.

## Discussion

The menuCH survey provides the first national meat consumption data, showing that the vast majority (90 %) of the Swiss population consumes meat. The mean amount of 109 g/d for all meat is lower than the published meat consumption of 131 g/d (48 kg/year) estimated from food balance sheets^(23)^, which often overestimate the consumption of many food groups. In food consumption surveys, individual food consumption is assessed. In contrast to food balance sheets, the available amount of food based on production and trade is calculated per capita of the population. Furthermore, food balance sheets do not account for food losses due to preparation, spoilage or food waste and do not consider meals eaten outside of the home^(24)^.

According to menuCH, the male population consumed almost twice as much meat as women, which was observed for all meat types except white meat. This sex-specific difference in meat consumption was significant and independent of total energy intake as reported in previous studies^(25–28)^. Lower meat consumption among women has been attributed to their greater interest in personal health, body weight maintenance and animal welfare concerns^(29)^; nevertheless, the perception of meat as a masculine food might be decreasing^(30)^.

Mean energy-standardised daily meat consumption was significantly higher in the French and Italian regions than in the German-language region. In recent studies using menuCH survey data, language region differences were also described for the consumption of other food groups and for overall diet quality^(16,31)^, and this is possibly linked to the influences of culinary habits in the neighbouring countries Germany, France and Italy. Nevertheless, energy-adjusted all meat consumption in France (absolute consumption not reported) and absolute mean daily all meat consumption in Germany and Italy were higher than mean daily all meat consumption in Switzerland^(26,32,33)^.

Our results show that UPM consumption was responsible for the higher all meat consumption in the French and Italian regions compared with the German-language region. Interestingly, in earlier Swiss Health Surveys, red meat was consumed more frequently in the German and in the French regions compared with the Italian language region^(34)^. However, the results are difficult to compare due to differences in the assessment methods. Comparisons of red, white and PM consumption amounts between countries are limited by differences in definitions and categorisations^(25)^. However, patterns of UPM and red meat consumption in the EPIC centres of France, Italy and Germany^(27)^ were indeed similar to those observed in the corresponding Swiss-language regions.

Poultry (without offal) was the highest consumed type of UPM by the Swiss population followed by beef, pork, veal, lamb and offal. According to food balance sheets, poultry consumption has increased since 2015, when the menuCH study was conducted^(10)^. Our result for poultry consumption (27 g/d) is comparable with that reported in France (26 g/d) but higher than amounts consumed in Italy (21 g/d) and Germany (18 g/d)^(26,35,36)^. Poultry is positively viewed for its high-quality protein, favourable lipid profile, as well as its vitamins and minerals^(37)^. Furthermore, it has neutral or protective associations with the major chronic diseases including obesity^(2,38)^. EPIC data show that beef, veal and lamb consumption was higher in the French and Italian centres than in the German centres, where pork consumption was higher^(27)^. In our results, pork consumption was highest in the German-language region, whereas beef, veal and lamb consumption was highest in the French and Italian-language regions. This could be due to the fact that traditional Italian and French meat dishes are prepared with different types of meat, for example, veal is widely used in Italian cuisine^(39)^, and lamb and offal are commonly used in French cuisine^(40)^.

The current study shows that the overweight and obese population in Switzerland consumed significantly higher amounts of all meat categories compared with the normal-weight population, independent of their energy intake, confirming results from Germany and France^(26,32)^. In an earlier study using menuCH Survey data, age, language region, education, income, household status, smoking, health status and physical activity were reported as determinants of meat consumption as well as for being overweight and obese^(41)^. Hence, high meat consumption could be an indicator of an unbalanced diet. Our results suggested one-parent families with children had higher meat consumption compared with couples without children, which did not agree with an earlier study in the USA^(42)^. In the German National Survey, the likelyhood of non-meat consumption was higher for smaller households^(26)^. Recent reports indicate that people with low or no meat consumption, not only in Germany and France but also in Switzerland, choose considerably healthier foods with regard to their nutrient and energy balance^(26,32,43)^.

Reductions in meat consumption are a concern in the ageing population, largely due to increased risk of nutritional inadequacy and higher requirements for some nutrients such as protein^(11)^. However, our analysis only shows reduced meat consumption (all meat, g/1000 kcal) in the 45–59 age category compared with the reference age group (30–44 years). In contrast, all meat and white meat consumption was higher in the youngest age group compared with the reference group, even though this age group was more likely to consume no meat in other studies^(26,44)^.

A significantly higher meat consumption, in particular white meat, was observed in the non-Swiss group, which is a highly diverse population group in Switzerland. Our results showed that participants with a tertiary-level education consumed less meat in general, which was consistent with results from Germany and France^(26,32)^, but this differed from the Swiss Health Survey findings that were based on meat consumption frequency^(34)^. We found that decreased red meat consumption was associated not only with a tertiary-level education, as reported in the French population^(45)^, but also with a primary level of education, most probably for different reasons^(13)^. For example, recent media attention on the risks of red meat would likely influence the meat choices of both low and highly educated participants, while the price of meat might have a greater influence on participants with a lower education. In our study, however, the association between meat consumption and income was NS. PM consumption was shown to be negatively associated with education in the Swiss population^(14)^, which is consistent with reports from Germany and France^(26,32)^. Participants living with their children consumed significantly more meat in general and more white meat compared with participants living with a partner but without children.

Dietary recommendations for meat consumption must consider both the benefits and risks of this food group and must address different demographic groups of the population. In Switzerland, it is recommended not to exceed more than 100–120 g of UPM, two to three days a week. The meat consumption data from the two 24HDR interviews provide no information about meat consumption frequency. However, 19 % of the menuCH participants exceeded the recommended UPM consumption amount of 120 g/d^(8)^. According to the Swiss Health Survey from 2017, 53 % of the population consumed an undefined amount of all meat including PM more than three times a week and therefore exceeded the frequency recommendations for meat consumption^(9)^.

A major strength of our study is the quantitative data derived from the two 24HDR interviews, which allowed the meat consumption (all types) of a representative national sample from the three main language regions of Switzerland to be assessed. Two 24HDR dietary recalls can be used to describe the habitual dietary intake distribution in food consumption surveys (i.e. for a population) given that they were collected on non-consecutive sampling days and cover all seasons and days of the week^(46)^. However, to accurately describe habitual meat consumption, the 24HDR interviews should be combined with a short FFQ to capture consumption of rarely eaten foods,^(47)^ in particular in combination with statistical models that take into account within-person variation, such as the Statistical Program to Assess Dietary Exposure^(48)^. Since the menuCH survey only included two 24HDR interviews, the assessment of meat consumption of those who only consume meat infrequently, e.g. flexitarians, is very limited. To better evaluate the links between meat consumption and potential health risks in Switzerland, future dietary surveys should also include markers of health such as blood pressure levels, blood samples and urine samples as collected in previous investigations in other countries^(49)^.

In conclusion, the current study is a comprehensive description of the first national data set on meat consumption in Switzerland, revealing that the mean daily consumption of red meat (37 g/d) was below the consumption level (50–100 g/d) that is considered to be associated with health risks^(2,4)^. However, the consumption of red meat differs between subgroups of the population and a considerable proportion of the population exceeds this recommended consumption level (29 % and 13 % of the population consumed more than 50 g/d and 100 g/d, respectively). Given the differences in health risks associated with red and white meat and their very different environmental impacts, it might be worthwhile to develop separate dietary recommendations for these meat categories, which take into account the latest findings on their effects on nutrition and health.

## Supplementary material

For supplementary material accompanying this paper visit https://doi.org/10.1017/S136898002200101X. click here to view supplementary material
