# Supplementary material for: Characterisation of meat consumption across socio-demographic, lifestyle and anthropometric groups in Switzerland: results from the National Nutrition Survey menuCH
Source: Public Health Nutr. 2022 Apr 25;25(11):3096–106. doi: 10.1017/S136898002200101X (PMC9991843; doi:10.1017/S136898002200101X)
Supplement: Supplementary file 1 [file S136898002200101Xsup001.docx]

**Supplementary Material**

Table S1: Mean daily consumption of unprocessed meat subcategories by total population, sex, language region and age group based on two 24-hour dietary recalls (g/1000 kcal, weighted,) ^1^ n = 2,057

|  | **Poultry** | | **Beef** | | **Pork** | | **Veal** | | **Lamb** | | **Offal** | | **Remaining Unprocessed Meat** | | |
| --- | --- | --- | --- | --- | --- | --- | --- | --- | --- | --- | --- | --- | --- | --- | --- |
|  | Mean  (g/1000 kcal) | SEM | Mean  (g/1000 kcal) | SEM | Mean  (g/1000 kcal) | SEM | Mean  (g/1000 kcal) | SEM | Mean  (g/1000 kcal) | SEM | Mean  (g/1000 kcal) | SEM | Mean  (g/1000 kcal) | SEM |  |
| **All CH** | 12.9 | 0.5 | 7.3 | 0.4 | 5.0 | 0.3 | 1.7 | 0.2 | 1.2 | 0.2 | 0.4 | 0.1 | 2.7 | 0.2 |  |
| **Gender** |  |  |  |  |  |  |  |  |  |  |  |  |  |  |  |
| Men | 13.4 | 0.9 | 8.0 | 0.6 | 5.6 | 0.5 | 1.9 | 0.3 | 1.3 | 0.2 | 0.6 | 0.1 | 3.9 | 0.5 |  |
| Women | 12.5 | 0.7 | 6.6 | 0.5 | 4.4 | 0.4 | 1.4 | 0.2 | 1.2 | 0.2 | 0.3 | 0.1 | 1.4 | 0.2 |  |
| **Language region^2^** |  |  |  |  |  |  |  |  |  |  |  |  |  |  |  |
| German | 11.5 | 0.6 | 5.6 | 0.4 | 5.4 | 0.4 | 1.8 | 0.2 | 1.0 | 0.2 | 0.2 | 0.1 | 2.1 | 0.2 |  |
| French | 15.4 | 1.1 | 11.1 | 0.9 | 4.0 | 0.5 | 0.9 | 0.3 | 2.2 | 0.4 | 1.0 | 0.2 | 4.2 | 0.7 |  |
| Italian | 19.6 | 2.4 | 10.8 | 1.8 | 4.7 | 1.2 | 3.3 | 0.8 | 0.2 | 0.2 | 0.2 | 0.2 | 2.6 | 0.6 |  |
| **Age group^3^** |  |  |  |  |  |  |  |  |  |  |  |  |  |  |  |
| 18-29 years | 17.4 | 1.4 | 6.1 | 0.8 | 4.1 | 0.7 | 1.2 | 0.3 | 0.9 | 0.3 | 0.1 | 0.1 | 3.8 | 0.8 |  |
| 30-44 years | 13.9 | 1.1 | 8.1 | 0.8 | 5.8 | 0.6 | 1.8 | 0.4 | 1.0 | 0.2 | 0.4 | 0.1 | 2.3 | 0.4 |  |
| 45-59 years | 12.1 | 0.9 | 7.4 | 0.7 | 5.1 | 0.5 | 1.8 | 0.3 | 0.9 | 0.3 | 0.6 | 0.2 | 3.1 | 0.5 |  |
| 60-75 years | 8.9 | 0.9 | 7.0 | 0.7 | 4.5 | 0.6 | 1.6 | 0.3 | 2.4 | 0.4 | 0.5 | 0.2 | 1.6 | 0.4 |  |

SEM, Standard error of the mean; ^1^weighted for sex, age, marital status, major area of Switzerland, household size, nationality, season, and weekday. ^2^ German-language region: cantons Aargau, Basel–Land, Basel–Stadt, Bern, Lucerne, St. Gallen, Zurich; French-language region: Geneva, Jura, Neuchâtel, Vaud; Italian-language region: Ticino. ^3^ Age corresponds to self-reported age on the first day of the 24-hour dietary recall interview. Remaining unprocessed meat includes unspecified unprocessed meat and meat from animals not specifically listed in this table.

All meat

Processed meat1

Unprocessed meat

Red meat

Mammals

Beef

Pork

Lamb

Mutton

Veal

Wild (venison)

Horse

Goat

Rabbit

Mammals offal

Unspecified red meat

White meat

Poultry

Chicken

Duck

Goose

Turkey

Pheasant

Guinea fowl

Ostrich

Unspecified white meat

Poultry offal

Unspecified poultry

Other UPM

Unspecified other UPM

Unprocessed meat

Poultry

Corresponding to poultry of categorisation 1 but without poultry offal

Beef

Pork

Lamb

Lamb

Mutton

Veal

Offal

Offal of all types of UPM

Remaining UPM

All UPM types of the categorisation 1 not listed here

Includes other UPM of categorisation 1
