# Supplementary material for: Characterisation of meat consumption across socio-demographic, lifestyle and anthropometric groups in Switzerland: results from the National Nutrition Survey menuCH
Source: Public Health Nutr. 2022 Apr 25;25(11):3096–106. doi: 10.1017/S136898002200101X (PMC9991843; doi:10.1017/S136898002200101X)
Supplement: Supplementary file 1 [file S136898002200101Xsup001.docx]

**Supplementary Material**

Table S1: Mean daily consumption of unprocessed meat subcategories by total population, sex, language region and age group based on two 24-hour dietary recalls (g/1000 kcal, weighted,) ^1^ n = 2,057

|  | **Poultry** | | **Beef** | | **Pork** | | **Veal** | | **Lamb** | | **Offal** | | **Remaining Unprocessed Meat** | | |
| --- | --- | --- | --- | --- | --- | --- | --- | --- | --- | --- | --- | --- | --- | --- | --- |
|  | Mean  (g/1000 kcal) | SEM | Mean  (g/1000 kcal) | SEM | Mean  (g/1000 kcal) | SEM | Mean  (g/1000 kcal) | SEM | Mean  (g/1000 kcal) | SEM | Mean  (g/1000 kcal) | SEM | Mean  (g/1000 kcal) | SEM |  |
| **All CH** | 12.9 | 0.5 | 7.3 | 0.4 | 5.0 | 0.3 | 1.7 | 0.2 | 1.2 | 0.2 | 0.4 | 0.1 | 2.7 | 0.2 |  |
| **Gender** |  |  |  |  |  |  |  |  |  |  |  |  |  |  |  |
| Men | 13.4 | 0.9 | 8.0 | 0.6 | 5.6 | 0.5 | 1.9 | 0.3 | 1.3 | 0.2 | 0.6 | 0.1 | 3.9 | 0.5 |  |
| Women | 12.5 | 0.7 | 6.6 | 0.5 | 4.4 | 0.4 | 1.4 | 0.2 | 1.2 | 0.2 | 0.3 | 0.1 | 1.4 | 0.2 |  |
| **Language region^2^** |  |  |  |  |  |  |  |  |  |  |  |  |  |  |  |
| German | 11.5 | 0.6 | 5.6 | 0.4 | 5.4 | 0.4 | 1.8 | 0.2 | 1.0 | 0.2 | 0.2 | 0.1 | 2.1 | 0.2 |  |
| French | 15.4 | 1.1 | 11.1 | 0.9 | 4.0 | 0.5 | 0.9 | 0.3 | 2.2 | 0.4 | 1.0 | 0.2 | 4.2 | 0.7 |  |
| Italian | 19.6 | 2.4 | 10.8 | 1.8 | 4.7 | 1.2 | 3.3 | 0.8 | 0.2 | 0.2 | 0.2 | 0.2 | 2.6 | 0.6 |  |
| **Age group^3^** |  |  |  |  |  |  |  |  |  |  |  |  |  |  |  |
| 18-29 years | 17.4 | 1.4 | 6.1 | 0.8 | 4.1 | 0.7 | 1.2 | 0.3 | 0.9 | 0.3 | 0.1 | 0.1 | 3.8 | 0.8 |  |
| 30-44 years | 13.9 | 1.1 | 8.1 | 0.8 | 5.8 | 0.6 | 1.8 | 0.4 | 1.0 | 0.2 | 0.4 | 0.1 | 2.3 | 0.4 |  |
| 45-59 years | 12.1 | 0.9 | 7.4 | 0.7 | 5.1 | 0.5 | 1.8 | 0.3 | 0.9 | 0.3 | 0.6 | 0.2 | 3.1 | 0.5 |  |
| 60-75 years | 8.9 | 0.9 | 7.0 | 0.7 | 4.5 | 0.6 | 1.6 | 0.3 | 2.4 | 0.4 | 0.5 | 0.2 | 1.6 | 0.4 |  |

SEM, Standard error of the mean; ^1^weighted for sex, age, marital status, major area of Switzerland, household size, nationality, season, and weekday. ^2^ German-language region: cantons Aargau, Basel–Land, Basel–Stadt, Bern, Lucerne, St. Gallen, Zurich; French-language region: Geneva, Jura, Neuchâtel, Vaud; Italian-language region: Ticino. ^3^ Age corresponds to self-reported age on the first day of the 24-hour dietary recall interview. Remaining unprocessed meat includes unspecified unprocessed meat and meat from animals not specifically listed in this table.
